# Supplementary material for: The microbiome biomarkers of pregnant women’s vaginal area predict preterm prelabor rupture in Western China
Source: Front Cell Infect Microbiol. 2024 Oct 31;14:1471027. doi: 10.3389/fcimb.2024.1471027 (PMC11560878; doi:10.3389/fcimb.2024.1471027)
Supplement: Supplementary file 1 [file DataSheet1.zip › compare_1/Community/Pieplot/species/group/C.species.pieplot.pdf]

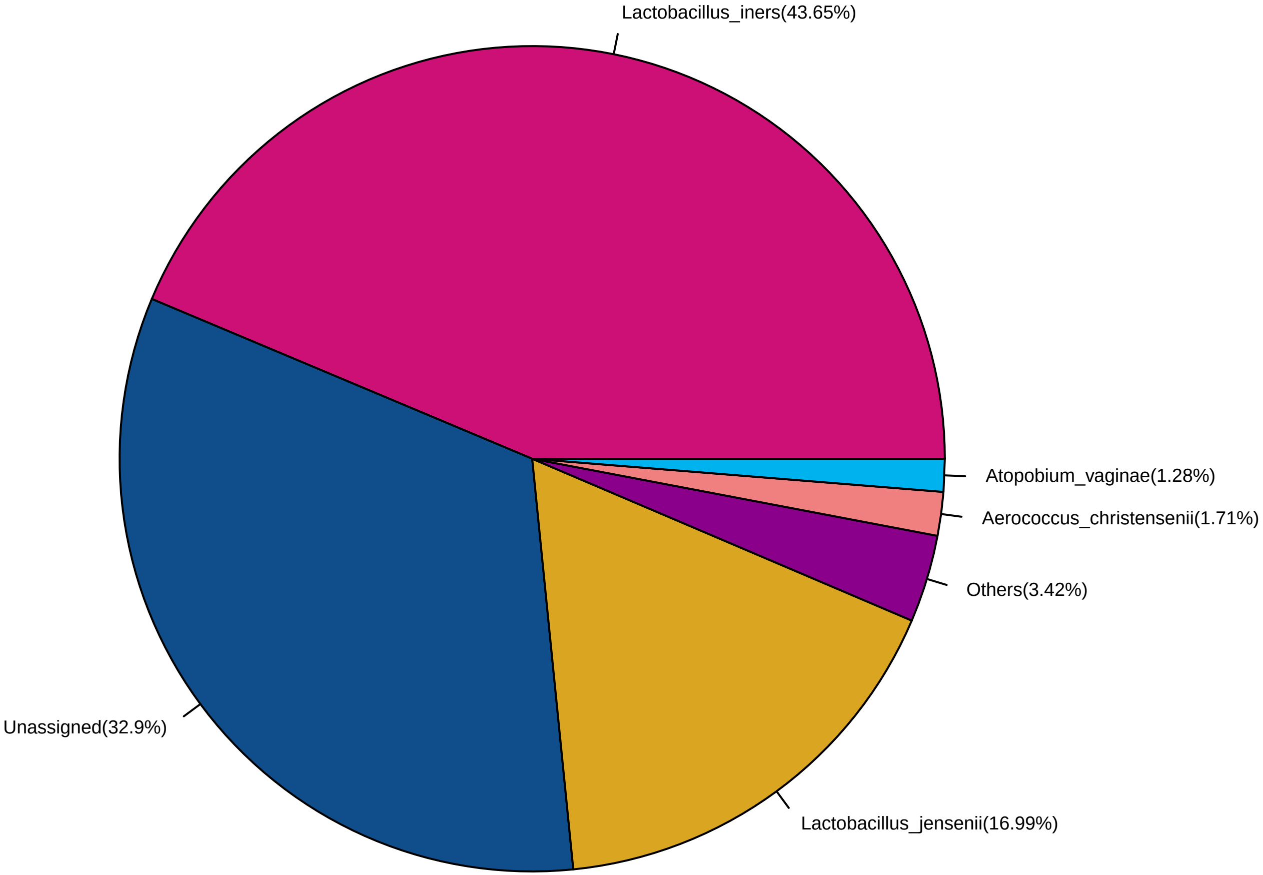

Lactobacillus_iners(43.65%)
Atopobium_vaginae(1.28%)
Aerococcus_christensenii(1.71%)
Others(3.42%)
Unassigned(32.9%)
Lactobacillus_jensenii(16.99%)
